# *In vitro* antimycobacterial and cytotoxic data on medicinal plants used to treat tuberculosis

**DOI:** 10.1016/j.dib.2016.03.088

**Published:** 2016-04-01

**Authors:** Joseph M. Nguta, Regina Appiah-Opong, Alexander K. Nyarko, Dorothy Yeboah-Manu, Phyllis G.A. Addo, Isaac D. Otchere, Abena Kissi-Twum

**Affiliations:** a Department of Clinical Pathology, Noguchi Memorial Institute for Medical Research, University of Ghana, Ghana; b Department of Public Health, Pharmacology and Toxicology, Faculty of Veterinary Medicine, University of Nairobi, Kenya; c Department of Bacteriology, Noguchi Memorial Institute for Medical Research, University of Ghana, Ghana; d Department of Animal Experimentation, Noguchi Memorial Institute for Medical Research, University of Ghana, Ghana

**Keywords:** *in vitro* antimycobacterial, *in vitro* cytotoxicity, Crude extracts, Tuberculosis, Medicinal plants, MTS assay, MABA assay

## Abstract

This article contains data on *in vitro* antimycobacterial activity and cytotoxicity of hydroethanolic crude extracts from five selected medicinal plant species traditionally used to treat tuberculosis in Ghanaian ethnomedicine, see “Medicinal plants used to treat TB in Ghana” [1]. The interpretation and discussion of these data and further extensive insights into drug discovery against tuberculosis from natural products of plant biodiversity can be found in “Antimycobacterial and cytotoxic activity of selected medicinal plant extracts” [2].

## Specification table

**Table:** 

Subject area	*Pharmacology and Toxicology*
More specific subject area	*Drug discovery against tuberculosis*
Type of data	*Tables*
How data was acquired	*in vitro antimycobacterial data was acquired using Microplate alamar blue assay (MABA), while cytotoxicity data was generated using MTS assay, with absorbance being read at 490 nm using Infinite M200 Pro*^*TM*^*plate reader (Tecan, Austria, GmbH)*
Data format	*Analyzed*
Experimental factors	*Test samples extracted with 80% ethanol by cold maceration, concentrated and lyophilized. Test crude extracts were dissolved in distilled water. Positive controls were prepared according to manufacturer׳s instructions*
Experimental features	*MABA was utilized to generate MIC values while MTS assay was used to evaluate cytotoxicity.*
Data source location	*Department of Clinical Pathology, Noguchi Memorial Institute for Medical Research, University of Ghana, Ghana and Department of Public Health, Pharmacology and Toxicology, Faculty of Veterinary Medicine, University of Nairobi, Kenya*
Data accessibility	*Data within this article*

## Value of the data

• This data provides a comprehensive *in vitro* antimycobacterial activity of hydroethanolic total extracts from five selected medicinal plants which can serve as a benchmark for other researchers.
• This data set includes *in vitro* cytotoxicity values from five selected ethnobotanicals and reference anti-TB drugs which can be used as a basis for further research.
• This data may provide insights for future drug development against tuberculosis.
• The data set can be used to guide further isolation of anti-TB compounds.
• This data set can be used for external validation of data acquired from similar experiments.

## 1 Data

Data on comparative antimycobacterial activity of total hydroethanolic extracts derived from five medicinal plant species is presented (Table 1). Data on *in vitro* cytotoxic activity of tested plant species and positive control drugs, isoniazid, rifampicin and ethambutol against MRC-5, human fetal lung fibroblast cell line (ATCC^®^ CCL-171™) is shared in Table 2.

## 2 Experimental design, materials and methods

### 2.1 Antibiotics and chemicals

Antimycobacterial reference standards [(ethambutol hydrochloride, European pharmacopoeia (EP) reference standard (EMB); Isoniazid >99%, (INH) and rifampicin 95% (RIF)] were all obtained from Sigma-Aldrich (St. Louis, MO) and stock solutions at 1000 μg/mL filter sterilized and stored at −20 °C until use. Middlebrook 7H9 broth supplemented with 0.05% (vol/vol) Tween 80 (Sigma); 10% OADC (oleic acid, albumin, dextrose, and catalase); Becton-Dickinson; 0.1% casitone and 0.5% glycerol was used to prepare working solutions at the following concentrations: RIF and EMB, 16 μg/mL; INH, 1.6 μg/mL and crude extracts, 10,000 μg/mL. A mixture of 10% Tween 80 and 10× Alamar blue dye (Alamar Biosciences/Accumed, Westlake, OH), at the ratio of 1:1 was prepared and stored at 4 °C for up to 1 week after sterilization by filtration.

### 2.2 Collection and preparation of plant materials

The rhizomes of *Zingiber officinale* and the leaves of *Chenopodium ambrosioides, Dissotis rotundifolia, Aloe vera var. barbadensis* and *Solanum torvum* were collected from the eastern region of Ghana, identified and prepared for bioassays as earlier described [1], [2].

### 2.3 Antimycobacterial assay

#### 2.3.1 Mycobacterial strains and growth conditions

Non-pathogenic fast growing mycobacterial strain, *Mycobacterium smegmatis* (ATCC®19420™), non-pathogenic slow growing strain, *Mycobacterium tuberculosis*; Strain H37Ra (ATCC^®^ 25177™) and slow growing pathogenic laboratory strain, *Mycobacterium tuberculosis subsp.tuberculosis* (ATCC^®^ 27294™) were obtained from American Type Culture Collection (Manassas, VA 20108, USA). The mycobacterial strains were maintained on Lowenstein–Jensen slopes and cultured on enriched media comprising of Middlebrook 7H9 broth (Difco, Detroit, MI) supplemented with 0.05% (vol/vol) Tween 80 (Sigma); 10% (vol/vol) OADC (oleic acid, albumin, dextrose, catalase; Difco), 1.0 g of casitone (Difco) per liter and 0.2% (vol/vol) glycerol (Sigma Chemical Co., Saint Louis, MO). The enriched culture medium was referred to as 7H9GC-Tween. Cultures were incubated at an aerobic atmosphere at 37 °C.

#### 2.3.2 Microplate alamar blue assay (MABA)

Mycobacterial strains were maintained on Lowenstein Jensen slopes, while the test inoculum was prepared in 7H9GC-tween broth, adjusted to a no. 1 McFarland tube standard, to give approximately 3.0×10^8^ cells and further diluted at the ration of 1:10 in the enriched broth (7H9GC-Tween) for anti-TB bioassays [2], [3]. The assay was conducted as earlier described by Nguta et al. [2] and Palomino et al. [3]. Briefly, 100 μL of the enriched broth was dispensed in each well of a sterile flat-bottom 96-well plate, followed by serial two-fold dilutions of the crude extracts. The positive control drugs were prepared directly in the microtiter plate. One hundred microliters (100 μL) of the prepared inoculum was added to each well. A sterile control comprising of the medium only and a growth control consisting of cells and growth medium were also included for each experiment. Evaporation during the incubation period was minimized by addition of sterile water to all perimeter wells of the 96-well microtiter plate. The covered microtiter plates were sealed in plastic bags and incubated at 37 °C under a normal atmosphere. After 7 days of incubation for *M. tuberculosis* H37Ra and *M. tuberculosis subsp. tuberculosis*, and 48 h of incubation for *M. smegmatis*, 30 μL of a mixture of 10% tween 80 and alamar blue solution in a ratio of 1:1 was added to each well, and the plate incubated overnight. A color change from blue to pink indicated mycobacterial growth, and the minimum inhibitory concentration (MIC) was taken as the highest dilution of the crude extract or positive control drug that prevented color change from blue to pink. The drug and crude extract concentration ranges used for the experiments were as follows: for positive controls, INH, 0.025–1.6 μg/mL, EMB and RIF, 0.25–16 μg/mL and for crude extracts, 10,000–19.5 μg/mL. All the tests were run in triplicate in a biosafety level three (BSL-3) laboratory.

### 2.4 in vitro cytotoxic evaluation

#### 2.4.1 Chemicals and reagents

Chemicals and reagents for cytotoxic investigations were procured from Promega (USA), Sigma (USA) and Gibco (USA).

#### 2.4.2 MRC-5 cell lines and culture conditions

Human fetal lung fibroblast cell line, MRC-5 (ATCC^®^ CCL-171™) was procured from the American Type Culture Collection (ATCC) (Manassas, VA 20108, USA). Cultures for cytotoxic evaluation were maintained in a phenol red free Dulbecco׳s modified essential medium/Ham׳s 12 nutrient mixture (DMEM/F12), (Gibco), supplemented with 1% (vol/vol) antibiotic [(2 mM L-glutamine, 100 U/mL Penicillin and 100 μg/mL Streptomycin; (Gibco)] and 5% [(vol/vol) fetal calf serum (JS Bioscience, Australia)]. MRC-5 fibroblast cells were incubated at 37 °C in a humidified 5% carbon dioxide incubator. The culture medium was removed from the flask and the cells were rinsed three times with sterile Hank׳s Balanced Salt Solution (HBSS), (Gibco), once the cells reached confluence. Trypsin/EDTA (Gibco, USA), was used to enzymatically remove the confluent layers, followed by suspension in culture medium. A light microscope (Leitz Wetzlar, Germany) was used to determine cell numbers after vital staining with trypan blue (0.4% (wt/vol); Sigma, USA).

#### 2.4.3 Preparation of test extracts

Total (crude) extracts were suspended in enriched phenol free Dulbecco׳s modified essential medium/Ham׳s 12 nutrient mixture (DMEM/F12), (Gibco) at the concentration of 10 000 μg/mL and dispersed by ultrasonic vibration for 15 min. Crude extracts were stirred on a vortex for one minute before every use to ensure uniform suspension.

#### 2.4.4 MTS assay

*in vitro* cytotoxicity assay was performed using the Promega CellTiter 96 AQueous Non-Radioactive Cell Proliferation (MTS) assay as earlier described [2], [4]. The procedure for cytotoxic evaluation was adopted from manufacturer׳s instructions and previously published papers ([5], [6]). Total extracts were suspended in enriched Dulbecco׳s modified essential medium/Ham׳s/12 nutrient mixture, followed by serial dilution across 96-well microtiter plates (100 μL), and incubation at 37 °C with 5% carbon dioxide (CO_2_) for a period of 24 h in a humidified CO_2_ incubator. Four (4 h) hours prior to the end of each experiment, an MTS mixture (20 μL/well) was added to each of the test wells of the 96-well microtiter plate. The plates were then placed on a microwell plate reader (Tecan, Austria, GmbH), shaken for 10 s and the absorbance of the formazan product read at 490 nm after the completion of each exposure period. Each experiment was run in triplicate. For each experiment, two internal controls were set: (i) IC_100_ consisting of medium only and (ii) an IC_0_ consisting of cells only. Background absorbance due to the non-specific reaction between the MTS reagent and the crude extracts was deducted from exposed cell values [7].

## Conflict of interest declaration

We declare no conflict of interest.

## Figures and Tables

**Table 1:** Comparative minimum inhibitory concentration (MIC in mg/mL) values of selected plant species against pathogenic and non-pathogenic mycobacterial strains.

	*Solanum torvum*	*Chenopodium ambrosioides*	*Dissotis rotundifolia*	*Zingiber officinale*	*Aloe vera var. barbadensis*	Isoniazid	Rifampicin	Ethambutol
*M. tb* H37Rv(ATCC^®^ 27294™)	1.25	>10	5	10	5	0.00003	0.00003	0.002
*M. smegmatis*(ATCC^®^ 19420™)	2.5	10	10	10	10	0.002	0.002	0.00025
*M. tb*; Strain H37Ra(ATCC^®^ 25177™)	0.1563	5	>10	2.5	2.5	0.00008	0.0005	0.0005

**Table 2:** Cytotoxic activity of selected medicinal plant species against human fetal lung fibroblast cell line, MRC-5 (ATCC^®^ CCL-171™).

Plant species	Concentration (mg/mL)	Absorbance 1	Absorbance 2	Absorbance 3	Mean	Corrected mean	Standard deviation	% cell viability	Blank	Negative control
*Solanum torvum*	0	0.2509	0.2509	0.2509	0.2509	0.1597	0.000	100.00	0.0865	0.2578
0.625	0.4418	0.4496	0.4434	0.4449	0.2076	0.004	130.01	0.0969	0.2687
1.25	0.4168	0.3984	0.3960	0.4037	0.0531	0.0110	33.27	0.0950	0.2651
2.5	0.6005	0.6030	0.5825	0.5953	0.1527	0.0110	95.64	0.0814	0.2674
5	0.9941	0.9438	0.9356	0.9578	0.3567	0.0320	223.38	0.0985	0.2696
10	1.4339	1.5394	1.4953	1.4895	0.1869	0.053	117.05	0.0980	0.2625
*Chenopodium ambrosioides*	0	0.2509	0.2509	0.2509	0.2509	0.1597	0.000	100.00	0.0895	0.1989
0.625	0.2614	0.2596	0.1789	0.2333	0.1011	0.0470	63.31	0.0957	0.2469
1.25	0.2678	0.2920	0.2117	0.2572	0.1209	0.0410	75.68	0.1566	0.2556
2.5	0.2947	0.3078	0.1921	0.2649	0.1324	0.0630	82.88	0.0912	0.2578
5	0.2986	0.3264	0.2005	0.2752	0.1232	0.066	77.12	0.1003	0.2372
10	0.1801	0.2119	0.1384	0.1768	−0.0162	0.0370	−10.14	0.091	0.2253
*Dissotis rotundifolia*	0	0.2509	0.2509	0.2509	0.2509	0.1597	0.000	100.00	0.087	0.2023
0.625	0.2994	0.3332	0.3428	0.3251	0.0762	0.0230	47.74	0.1063	0.2603
1.25	0.3127	0.3252	0.3503	0.3294	0.0150	0.019	9.39	0.1	0.2690
2.5	0.2960	0.3342	0.3421	0.3241	−0.0405	0.025	−25.36	0.09	0.2728
5	0.4618	0.4840	0.5069	0.4842	−0.0603	0.023	−37.74	0.1042	0.2676
10	0.7912	0.7025	0.8740	0.7892	−0.2213	0.0860	−138.5	0.0876	0.2699
*Zingiber officinale*	0	0.2509	0.2509	0.2509	0.2509	0.1597	0.000	100.00	0.0979	0.0912
0.625	0.2692	0.2796	0.2649	0.2712	0.1322	0.008	82.80	0.0892	0.1390
1.25	0.3164	0.3387	0.2655	0.3069	0.1732	0.038	108.43	0.0880	0.1337
2.5	0.1681	0.1577	0.1858	0.1705	0.0216	0.014	13.55	0.0788	0.1489
5	0.1874	0.2249	0.2719	0.2281	0.0311	0.042	19.45	0.0879	0.1970
10	0.3833	0.4711	0.4020	0.4188	0.1590	0.0460	99.56	0.0942	0.2598
*Aloe vera var. barbadensis*	0	0.2509	0.2509	0.2509	0.2509	0.1597	0.000	100.00	0.0888	0.0912
0.625	0.2902	0.2886	0.2770	0.2853	0.1269	0.007	79.44	0.0854	0.1584
1.25	0.2863	0.2773	0.2625	0.2754	0.1389	0.0120	86.95	0.0916	0.1365
2.5	0.3027	0.2891	0.2806	0.2908	0.1258	0.0110	78.77	0.0812	0.1650
5	0.1907	0.1852	0.1938	0.1899	−0.0148	0.004	−9.27	0.0845	0.2047
10	0.1964	0.2024	0.2019	0.2002	−0.0319	0.003	−19.95	0.0893	0.2321
Isoniazid	0	0.3547	0.3547	0.3547	0.3547	0.1427	0.000	100.00	0.2136	0.2120
0.625	0.4501	0.3552	0.3296	0.3783	0.1569	0.0630	109.95	0.2117	0.2214
1.25	0.3441	0.3282	0.3565	0.3429	0.1214	0.0140	85.097	0.2064	0.2215
2.5	0.3815	0.3882	0.3476	0.3724	0.1533	0.0220	107.45	0.2097	0.2191
5	0.3327	0.4934	0.3431	0.3897	0.1748	0.0900	122.51	0.2279	0.2149
10	0.4652	0.3455	0.3621	0.3909	0.1701	0.0650	119.22	0.2303	0.2208
Rifampicin	0	0.3547	0.3547	0.3547	0.3547	0.1427	0.000	100.00	0.2139	0.2120
0.625	0.4120	0.3482	0.3352	0.3651	0.1468	0.041	102.897	0.2089	0.2183
1.25	0.3450	0.4193	0.3511	0.3718	0.0936	0.041	65.592	0.2098	0.2782
2.5	0.3597	0.3825	0.3529	0.3650	0.1033	0.0160	72.413	0.2085	0.2617
5	0.3514	0.3610	0.3770	0.3631	0.1268	0.013	88.881	0.2070	0.2363
10	0.3859	0.3830	0.4586	0.4092	0.1606	0.043	112.52	0.2319	0.2486
Ethambutol	0	0.3547	0.3547	0.3547	0.3547	0.1427	0.000	100.00	0.2025	0.2120
0.625	0.3909	0.3756	0.3550	0.3738	0.1557	0.0180	109.133	0.2128	0.2181
1.25	0.3380	0.3549	0.3442	0.3457	0.1231	0.009	86.265	0.18112	0.2226
2.5	0.3498	0.3287	0.3309	0.3365	0.1249	0.0120	87.503	0.2132	0.2116
5	0.3447	0.3696	0.3254	0.3466	0.1354	0.0220	94.861	0.1739	0.2112
10	0.3511	0.3396	0.3304	0.3404	0.1037	0.0100	72.647	0.2096	0.2367
