# Supplementary material for: In vitro antimycobacterial and cytotoxic data on medicinal plants used to treat tuberculosis
Source: Data Brief. 2016 Apr 1;7:1124–30. doi: 10.1016/j.dib.2016.03.088 (PMC4833128; doi:10.1016/j.dib.2016.03.088)
Supplement: Supplementary file 1 — Supplementary material [file mmc1.doc]

**Conflict of interest declaration**

We declare no conflict of interest.
